# Intraoperative molecular imaging of colorectal lung metastases with SGM-101: a feasibility study

**DOI:** 10.1007/s00259-023-06365-3

**Published:** 2023-08-08

**Authors:** Ruben P. J. Meijer, Hidde A. Galema, Robin A. Faber, Okker D. Bijlstra, Alexander P. W. M. Maat, Françoise Cailler, Jerry Braun, Stijn Keereweer, Denise E. Hilling, Jacobus Burggraaf, Alexander L. Vahrmeijer, Merlijn Hutteman, On behalf of the SGM-CLM study group, Mats I. Warmerdam, Feredun Azari, Sunil Singhal, Dima D. A. Almandawi, Edris A. F. Mahtab, Ghada M. M. Shahin, Michail Doukas, Cornelis Verhoef, Bérénice Framery

**Affiliations:** 1 Department of Surgery, Leiden University Medical Center, Albinusdreef 2, 2333 ZA Leiden, The Netherlands; 2 Center for Human Drug Research, Zernikedreef 8, 2333 CL Leiden, The Netherlands; 3 Department of Surgical Oncology and Gastrointestinal Surgery, Erasmus MC Cancer Institute, Doctor Molewaterplein 40, 3015 GD Rotterdam, The Netherlands; 4 Department of Otorhinolaryngology, Head and Neck Surgery, Erasmus MC Cancer Institute, Doctor Molewaterplein 40, 3015 GD Rotterdam, The Netherlands; 5 Department of Cardiothoracic Surgery, Erasmus Medical Center, Doctor Molewaterplein 40, 3015 GD Rotterdam, The Netherlands; 6 Surgimab, 10 Parc Club du Millénaire, 1025 Avenue Henri Becquerel, 34000 Montpellier, France; 7 Department of Cardiothoracic Surgery, Leiden University Medical Center, Albinusdreef 2, 2333 ZA Leiden, The Netherlands; 8 Department of Thoracic Surgery, Hospital of the University of Pennsylvania, 3400 Civic Center Boulevard, 14Th Floor, South Pavilion, Philadelphia, PA 19104 USA; 9 Department of Surgery, Radboud University Medical Center, Geert Grooteplein Zuid 10, GA 6525 Nijmegen, The Netherlands; 10 Department of Pathology, Erasmus Medical Center, Doctor Molewaterplein 40, GD 3015 Rotterdam, The Netherlands

**Keywords:** SGM-101, Colorectal lung metastases, Carcinoembryonic antigen, CEACAM5, Molecular imaging, Near-infrared fluorescence imaging

## Abstract

**Purpose:**

Metastasectomy is a common treatment option for patients with colorectal lung metastases (CLM). Challenges exist with margin assessment and identification of small nodules, especially during minimally invasive surgery. Intraoperative fluorescence imaging has the potential to overcome these challenges. The aim of this study was to assess feasibility of targeting CLM with the carcinoembryonic antigen (CEA) specific fluorescent tracer SGM-101.

**Methods:**

This was a prospective, open-label feasibility study. The primary outcome was the number of CLM that showed a true positive fluorescence signal with SGM-101. Fluorescence positive signal was defined as a signal-to-background ratio (SBR) ≥ 1.5. A secondary endpoint was the CEA expression in the colorectal lung metastases, assessed with the immunohistochemistry, and scored by the total immunostaining score.

**Results:**

Thirteen patients were included in this study. Positive fluorescence signal with in vivo, back table, and closed-field bread loaf imaging was observed in 31%, 45%, and 94% of the tumors respectively. Median SBRs for the three imaging modalities were 1.00 (IQR: 1.00–1.53), 1.45 (IQR: 1.00–1.89), and 4.81 (IQR: 2.70–7.41). All tumor lesions had a maximum total immunostaining score for CEA expression of 12/12.

**Conclusion:**

This study demonstrated the potential of fluorescence imaging of CLM with SGM-101. CEA expression was observed in all tumors, and closed-field imaging showed excellent CEA specific targeting of the tracer to the tumor nodules. The full potential of SGM-101 for in vivo detection of the tracer can be achieved with improved minimal invasive imaging systems and optimal patient selection.

**Trial registration:**

The study was registered in ClinicalTrial.gov under identifier NCT04737213 at February 2021.

**Supplementary information:**

The online version contains supplementary material available at 10.1007/s00259-023-06365-3.

## Introduction

Around 5% of the patients with colorectal cancer (CRC) develop lung metastases after treatment with curative intent [1, 2]. For selected, oligo-metastatic patients, metastasectomy is an important treatment option so long as the primary disease is under control. Tumor identification during metastasectomy is sometimes challenging, as nodules can be small. Positive margins are associated with decreased overall survival, which makes complete removal of the tumor of utmost importance [3]. While the introduction of video-assisted thoracic surgery (VATS) has reduced surgical morbidity, tumor identification has become more challenging. Therefore, interest is growing in other methods for intraoperative detection of colorectal lung metastases (CLM).

Intraoperative, tumor-specific, near-infrared (NIR) fluorescence imaging is developed for several surgical procedures, including lung surgery [4]. To realize NIR fluorescence tumor imaging, patients are administered intravenously with a tumor-specific tracer attached to a fluorophore. Imaging of these agents with a fluorescence imaging system allows for real-time intraoperative visualization of the tumor [5]. SGM-101 is a fluorescent tracer that consists of a monoclonal antibody that targets carcinoembryonic antigen (CEA), labeled with a NIR fluorophore (BM-104). This fluorophore has an excitation and emission wavelength around 700 nm [6]. CEA is overexpressed in > 95% of the colorectal cancers and thus an excellent target for molecular imaging of CRC [7]. NIR fluorescence imaging of CLM with SGM-101 may improve intraoperative detection of these tumors and thus increase the chance of a complete resection of the tumor.

Intraoperative NIR fluorescence imaging with SGM-101 has been studied in trials for locally advanced CRC, peritoneal metastases of CRC, colorectal liver metastases, and pancreatic cancer [8–12]. In a phase II rectal cancer trial, NIR fluorescence imaging with SGM-101 resulted in a change in surgical plan in 7 out of 37 patients. Currently, two phase III trials are ongoing with SGM-101 for CRC and peritoneal metastases [13, 14]. The aim of this study was to assess the potential of targeting CLM with SGM-101.

## Methods

This study was reviewed and approved by the medical ethical committee “Leiden-Den Haag-Delft” and conducted according to the declaration of Helsinki (10th version, Fortaleza, 2013). Informed consent was obtained from all study participants. The study was registered in Clinicaltrials.gov under identifier NCT04737213. The study was conducted in the Leiden University Medical Center (LUMC) and the Erasmus MC Cancer Institute (EMC).

### Study design

This was a prospective, open-label, non-randomized feasibility study to assess the ability of SGM-101 to target CLM. In this single arm trial, all patients were intravenously administered with SGM-101. SGM-101 was supplied by Surgimab (Montpellier, France). All patients received intravenous administration three to five days prior to surgery, based on earlier study protocols [10–12]. The drug was administered over 30 min, and patients were observed for 3 h after infusion. Prior to resection, in vivo fluorescence imaging was performed to delineate the tumor and to assess for possible occult lesions. After resection of the tumor, in vivo fluorescence imaging of the wound bed and ex vivo imaging of the resected specimen was performed to assess for possible tumor positive resection margins. Patients at least 18 years old, scheduled for resection of (suspected) CLM, and willing and able to give written informed consent were eligible for inclusion. Exclusion criteria were history of any anaphylactic reaction, other malignancies either currently active or diagnosed in the last 5 years, hepatic or renal insufficiencies, blood count abnormalities, known positive test for HIV, hepatitis B surface antigen or hepatitis C virus antibody or patients with untreated serious infections, patients pregnant or breastfeeding, or any condition that the investigator considered to be potentially jeopardizing the patient’s wellbeing or the study objectives.

### Outcomes

The primary outcome of this study was the number of CLM that showed a true positive fluorescence signal with SGM-101 and a NIR fluorescence imaging system. Secondary endpoints were the optimal dose of SGM-101 for fluorescence imaging of CLM, possible change in surgical management based on fluorescence imaging, and concordance between fluorescence imaging and CEA expression on the corresponding tissue slides.

For the primary outcome, lesions were considered fluorescent (i.e., a positive index test) if the signal-to-background ratio (SBR) was ≥ 1.5 [15]. The reference standard for demonstrating CLM was final histopathological assessment. Imaging of the CLM was performed in three settings: in vivo imaging, ex vivo imaging of the whole specimen on the back table (back table imaging), and ex vivo imaging of bread loaf slides in a closed-field imaging device (closed-field imaging). In vivo and back table imaging was performed with the Quest spectrum V2 fluorescence camera (Middenmeer, The Netherlands). In vivo and ex vivo imaging was performed intraoperatively and could therefore potentially affect the surgical procedure. During VATS, the endoscopic camera of Quest spectrum V2 was used. Closed-field imaging was performed with the PEARL MSI imaging system (Li-Cor, Lincoln, Nebraska, USA). Closed-field imaging of the tissue bread loaves was performed on the day after surgery and could therefore not affect the surgical procedure. SBRs were calculated with the “Quest TBR tool” (Quest Medical Imaging, Middenmeer, The Netherlands) and Image Studio software (Li-Cor, Lincoln, Nebraska, USA). The SBR was defined as the mean fluorescence intensity of the signal derived from the tumor divided by the mean fluorescence intensity of signal derived from the surrounding normal tissue.

Doses of 7.5, 10, and 12.5 mg were studied. The optimal dose was decided based on closed-field bread loaf imaging. As this was a feasibility study, no direct change in surgical management was performed based on intraoperative fluorescence imaging alone. However, possible change in surgical management was noted as a secondary outcome measure (type D study [16]). CEA expression was assessed by immunohistochemistry with the monoclonal mouse antibody against CEACAM5 (clone number CI-P83-1, Santa Cruz Biotechnology) [12]. Scoring of staining was done by multiplying the intensity score and the proportion score, to calculate the total immunostaining score (TIS) [17]. A dedicated pathologist (MD) performed scoring of the immunohistochemistry-stained tissue slides.

### Statistics

R software (version 4.1.0, R Foundation for statistical computing, Vienna, Austria) was used for statistical analysis. Numerical data was described with median and interquartile range (IQR) or range. To assess SBR differences between dose groups, a Kruskal–Wallis test was performed. To assess the influence of overlying lung parenchyma on fluorescence signal intensity, tumors were categorized as closer or further distanced than 14 mm of the visceral pleura as defined by pre-operative computed tomography (CT) [18]. *P* < 0.05 was considered significant. The sample size is based upon experience with this type of compounds and not on a formal power calculation. Using the 3 + 3 dose escalation design, a minimum of 9 and a maximum of 15 patients will be included, corresponding to a minimum of 3 patients per dose level. Patients were allocated in a chronological order.

## Results

Between January 2021 and September 2022, 13 patients (ten males, three females) with a median age of 56 years (IQR: 54.5–66.5) were included. Patient and surgical characteristics are described in Table 1. There were no (serious) adverse events with any possible relationship to the administration of SGM-101.

**Table 1:** Patient- and surgical characteristics

		*n* (%)*
Patients		13 (100)
Sex	*Male*	10 (77)
	*Female*	3 (23)
Hospital	*LUMC*	7 (46)
	*EMC*	6 (54)
Age (median [IQR])		56 [54.5–66.5]
Serum CEA (μg/ml) (median [IQR])		5.8 [3.33–8.5]
Smoking history	*Current*	1 (8)
	*Former*	4 (31)
	*Never*	8 (62)
Location metastasis	*Right upper lobe*	5 (28)
	*Middle lobe*	1 (6)
	*Right lower lobe*	6 (33)
	*Left upper lobe*	3 (17)
	*Left lower lobe*	3 (17)
Surgical procedure**	*Lobectomy*	4 (31)
	*Segment resection*	2 (15)
	*Wedge resection*	9 (69)
	*Lymphadenectomy*	5 (38)
Surgical approach	*Thoracotomy*	2 (15)
	*VATS*	9 (70)
	*RATS*	2 (15)***

^*^Percentages may not always add up to 100 due to rounding to full numbers

^**^Multiple patients underwent combined lobectomy and wedge/segment resections

^***^One converted to thoracotomy due to hemorrhage

*Abbreviations: LUMC* Leiden University Medical Centre*, EMC* Erasmus Medical Centre*, IQR* interquartile range*, VATS* video-assisted thoracic surgery*, RATS* robot-assisted thoracic surgery

### Tumor lesions

**Fig. 1:**
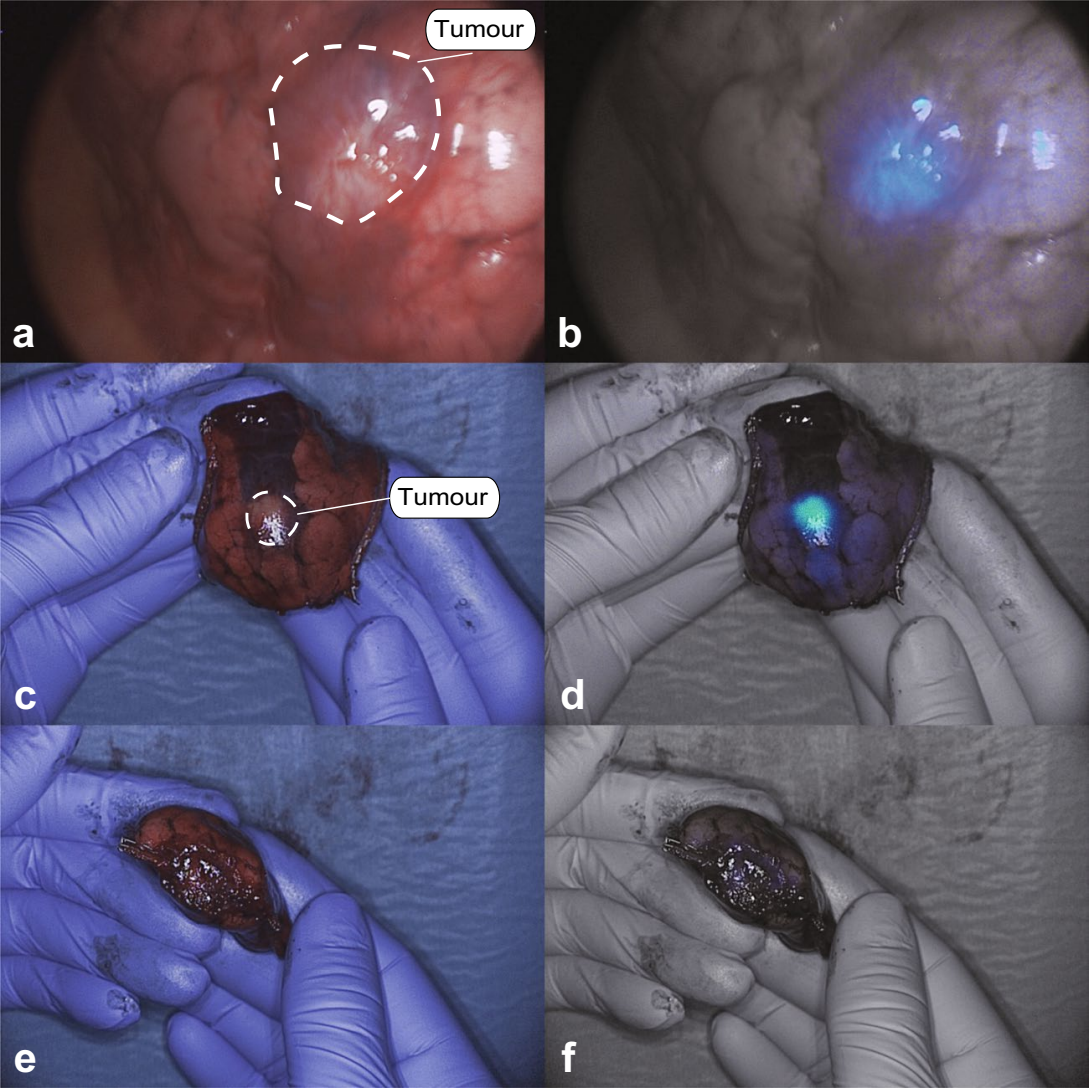
White light (left row) and gradient fluorescence overlay (right row) in vivo (**a**, **b**) and back table (**c**, **d**, **e**, **f**) images of lesion 7

Eighteen CLM were resected. Characteristics of all lesions are described in Table 2. In vivo imaging was performed on 16 lesions, back table imaging on 15 lesions, and closed-field imaging on 18 lesions. A positive fluorescence signal was observed in five lesions (31%) in vivo*,* in seven lesions (47%) with back table imaging and in 17 lesions (94%) with the closed-field imaging. Median SBRs for the three imaging modalities were 1.00 (IQR: 1.00–1.53), 1.45 (IQR: 1.00–2.05), and 4.81 (IQR: 2.70–7.41) respectively. All metastases were detected based on preoperative imaging and white light inspection. No lesions were identified solely based on NIR fluorescence imaging. Five metastases were located > 14 mm of the pleura, none of which showed positive in vivo fluorescence (median SBR: 1.00, range 1.00–1.34). For lesions ≤ 14 mm of the pleura, 5 out of 11 (45%) were fluorescent in vivo (median SBR: 1.34, range: 1.00–2.15) and 6 out of 11 (64%) lesions on the back table (median SBR: 1.98, range 1.00–3.53). Figure 1 presents an example of in vivo and back table imaging (lesion 7).

**Table 2:** Characteristics per lesion

Preoperative				Intraoperative					Pathology				IHC		
ID	Dose SGM-101	Lesion	Location	Distance to pleura (mm)	CT	WL	TBR (in vivo)*	TBR (ex vivo)*	Histopathology	Margin (mm)	TBR (bread loaf)*	Concordance **	**IS**	**PS**	**TIS**
1	7.5	1	RLL	6	+	+	2.15	Miss	Metastasis crc	4	6.1	TP	3	4	12
		2	Station 11	n/a	-	+	1	Miss	Benign LN	n/a	Miss	TN	n/a	n/a	n/a
		3	RLL	17	+	-	1.34	Miss	Metastasis crc	20	5.91	TP	3	4	12
2	7.5	4	RUL	10	+	+	1.62	3.53	Metastasis crc	5	6.43	TP	3	4	12
3	7.5	5	LLL	0	+	+	1.51	1.98	Metastasis crc	10	4.09	TP	3	4	12
		6	LLL	18	+	+	1	1.57	Metastasis crc	23	5.07	TP	3	4	12
4	10	7	RLL	22	+	+	Miss	Miss	Metastasis crc	2	10.44	TP	3	4	12
5	10	8	LUL	0	+	+	1.61	2.19	Metastasis crc	free	4.54	TP	3	4	12
6	10	9	RUL	8	+	+	1	1	Fibrosis	n/a	Miss	TN	n/a	n/a	n/a
		10	RUL	2	+	+	1	1	Fibrosis	n/a	Miss	TN	n/a	n/a	n/a
		11	RUL	n/a	-	+	1	1	Fibrosis	n/a	Miss	TN	n/a	n/a	n/a
		12	RLL	23	+	+	1	1	Metastasis crc	3	1.52	TP	3	4	12
		13	ML	0	+	+	1	1	Metastasis crc	5	2.35	TP	3	4	12
7	12.5	14	RLL	3	+	+	1.34	1.19	Metastasis crc	6	2.99	TP	3	4	12
		15	ML	1	+	+	1	1	Metastasis crc	1	2.01	TP	3	4	12
		16	RUL	15	+	+	1	1	Metastasis crc	10	1.23	FN	3	4	12
		17	Station 11	n/a	-	+	Miss	1	Benign LN	n/a	Miss	TN	n/a	n/a	n/a
8	12.5	18	RUL	9	+	+	Miss	1.45	Metastasis crc	26	9.93	TP	3	4	12
9	12.5	19	RLL	7	+	-	1	2.04	Metastasis crc	free	3.8	TP	3	4	12
10	7.5	20	LUL	20	+	+	1	1	Metastasis crc	10	8.06	TP	3	4	12
11	7.5	21	LUL	10	+	+	1	2.13	Metastasis crc	free	9.88	TP	3	4	12
12	12.5	22	RUL	2	+	+	1	1.37	Metastasis crc	free	2.49	TP	3	4	12
13	10	23	LLL	0	+	+	1.6	2.06	Metastasis crc	free	7.73	TP	3	4	12
		24	Station 7	n/a	-	+	Miss	1	Benign LN	n/a	Miss	TN	0	0	0
		25	Station 8	n/a	-	+	Miss	1.78	Malignant LN	n/a	Miss	TP	3	4	12
		26	Station 9	n/a	-	+	Miss	1	Benign LN	n/a	Miss	TN	n/a	n/a	n/a
		27	Station 10	n/a	+	+	Miss	1.63	Malignant LN	n/a	Miss	TP	3	4	12
		28	Station 11 ventral	n/a	+	+	1.59	1.69	Malignant LN	n/a	Miss	TP	3	4	12
		29	Station 11 dorsal	n/a	-	+	Miss	1	Benign LN	n/a	Miss	TN	n/a	n/a	n/a

^*^A TBR of ≥ 1.5 is considered fluorescence positive, **concordance between fluorescence imaging and histopathology

*Abbreviations: CT* Computed tomography, *WL* white light suspect, *TBR* tumor-to-background ratio, *n/a* not applicable, *IS* intensity score, *PS* proportion score, *TIS* total immunostaining score*, miss* missing*, LN* lymph node*, crc* colorectal cancer, *TP* true positive, *TN* true negative, *FN* false negative

### Lymph nodes

In patients 1 and 7, two benign lymph nodes were resected based on white light suspicion, but were fluorescence-negative (true negatives). In patient 13, a lymphadenectomy was performed for preoperatively identified hilar lymph node metastases. Three malignant lymph nodes were fluorescent on the back table (lesions 25, 27, 28, true positives). Three other non-fluorescent lesions were resected based on clinical suspicion for tumor involvement. All three contained fibrosis without tumor (lesions 24, 26, 29, true negatives). Supplemental Fig. 1 presents the white light and gradient overlay fluorescence images of three lymph nodes (lesions 25, 26, 28).

### SGM-101 dose

**Fig. 2:**
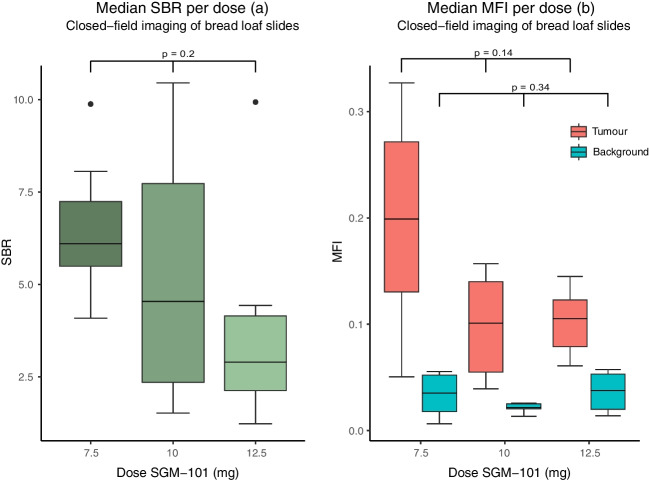
The signal-to-background ratios per dose group (**a**) and the mean fluorescence intensities (MFI) for tumor and background tissue per dose group (**b**). The boxes represent medians with q1 and q3 and the error bars represent the range

Five patients (seven lesions) were injected with 7.5 mg SGM-101, four patients (five lesions) with 10 mg, and four patients (six lesions) with 12.5 mg. Median SBRs (closed-field imaging) for the dose levels were 6.1 (IQR: 5.50–7.25), 4.54 (IQR: 2.35–7.73), and 2.9 (IQR: 2.13–4.25) respectively (Fig. 2a, p = 0.20). There was no difference in absolute tumor or background mean fluorescence intensity (MFI) between the three dose groups (tumor: *p* = 0.14, background: *p* = 0.34, Fig. 2b).

### Potential change in surgical management and clinical outcome

**Fig. 3:**
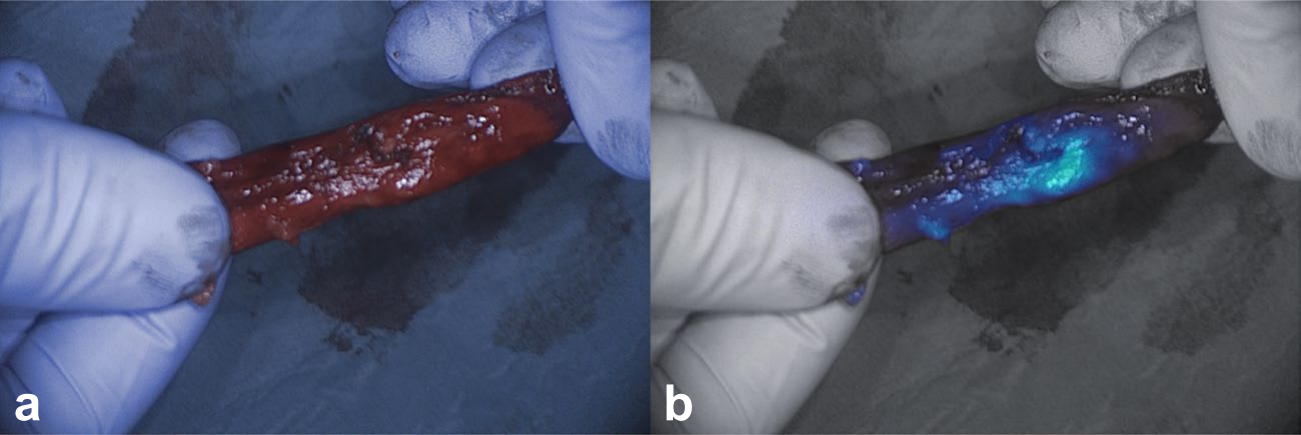
White light (**a**) and gradient fluorescence overlay (**b**) images of an invisible and non-palpable tumor with a clear fluorescence signal (lesion 19)

In one patient, three clinically suspect, non-fluorescent nodules were resected (true negatives, lesions 9, 10, 11). In patient 5, the surgeon was unsure whether a complete removal of the tumor was achieved. Therefore, a small additional resection was performed. Fluorescence back table imaging of the primary specimen showed no suspicion of tumor involvement in the resection margin (Fig. 1e and f). Final pathology assessment of primary resected specimen confirmed absence of tumor in the resection margin. In patient 9, tumor identification was based on the location on the CT scan and white light inspection. After resection, it was unclear whether the tumor was in the specimen, as the nodule was not palpable. After removing the staples, a clear fluorescent signal was observed in the specimen (Fig. 3). The fluorescent tissue was sent for frozen section analysis and confirmed as malignant. Resection margin assessment by in vivo wound bed imaging and ex vivo imaging of the resected specimen did not reveal any suspect tumor positive resection margins in any of the patients, which was confirmed by final histopathology.

### CEA expression

**Fig. 4:**
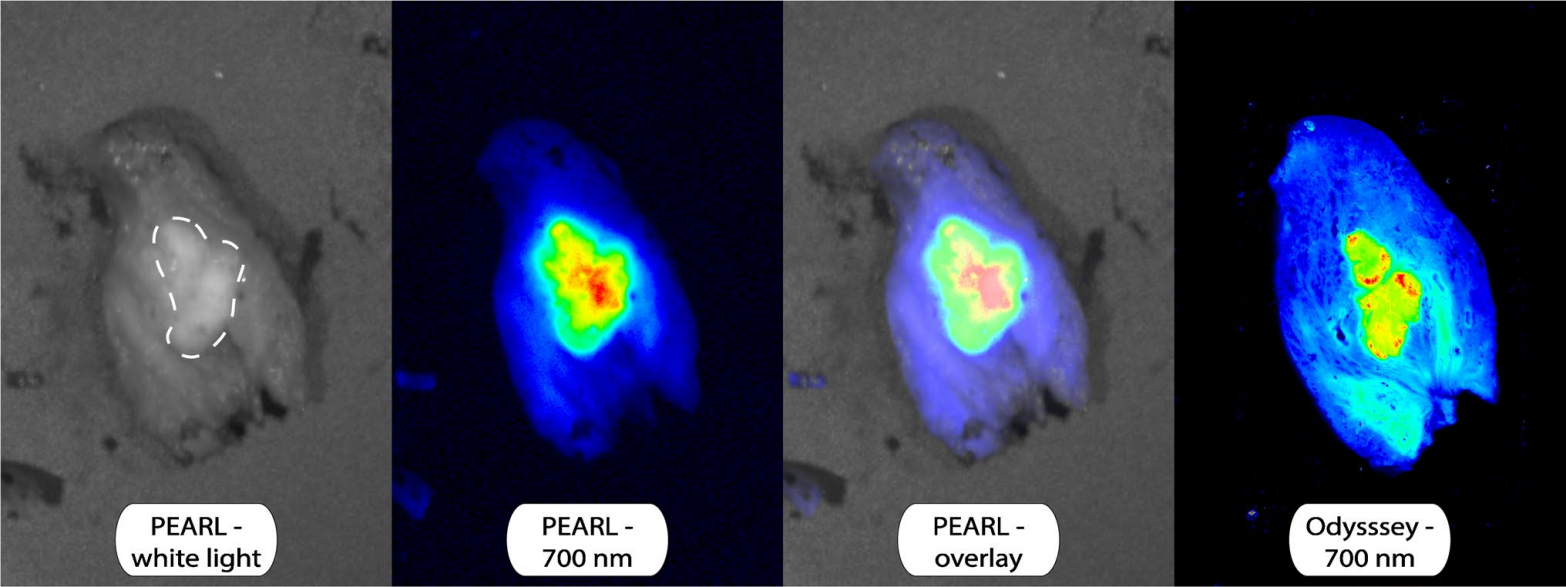
A tissue slide of a CLM imaged with the PEARL MSI and Odyssey CLx scanner

**Fig. 5:**
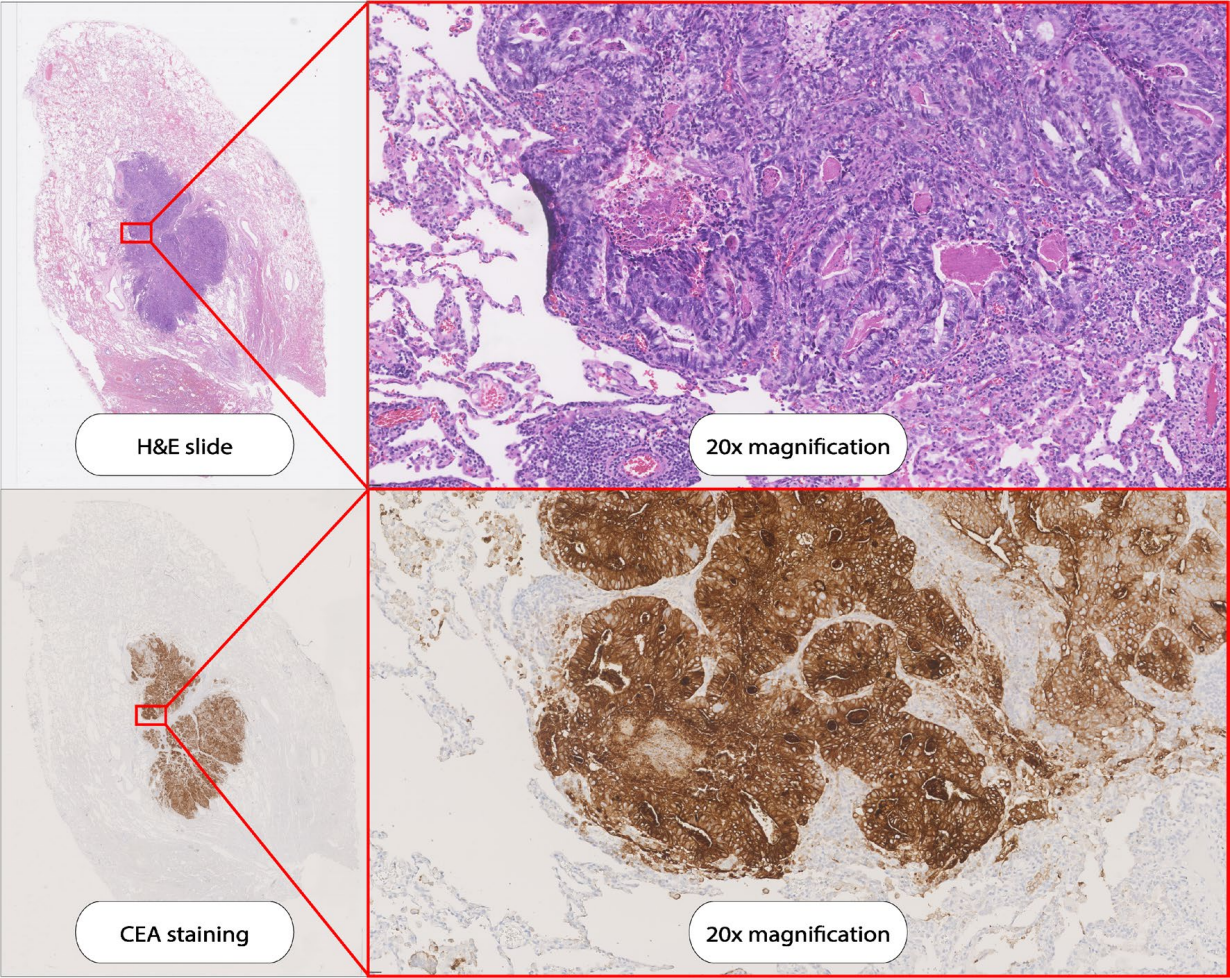
Hematoxylin and eosin (HE) staining and carcinoembryonic antigen (CEA) immunohistochemistry staining on a the tissue slide as demonstrated in Fig. 4

Preoperative serum CEA levels were elevated (> 5.0 μg/L) in 6 out of 11 patients and unknown in the other two patients. CEA expression of all 18 tumor lesions was assessed by immunohistochemistry and all 18 lesions had a total immunostaining score (TIS) of 12 out of 12. Figure 4 presents a bread loaf tissue section of a CLM imaged with several imaging modalities. Figure 5 presents a slide from the same tissue block with the H&E and CEA immunohistochemistry staining. Three tumor containing lymph nodes had maximum CEA expression (TIS: 12). One normal control lymph node had no CEA expression (lesion 24, TIS: 0). CEA expression per lesion is shown in Table 2.

## Discussion

The present study shows that targeting of SGM-101 to CLM was accurate and that CEA is the target of choice for tumor specific imaging of CLM. Challenges remain with in vivo detection of the tumor lesions, especially with the minimally invasive NIR fluorescence imaging system. The full potential of SGM-101 for in vivo detection of the tracer may therefore be achieved with improved minimally invasive imaging systems. Optimal patient selection may also further improve the efficacy of SGM-101. If intraoperative identification of the lesion is expected to be challenging, SGM-101 may help for the detection of superficial lesions. Identification of lesions deeper in the lung parenchyma is not expected to be possible with the technique, as overlying lung tissue negatively affects the observed fluorescence signal. An earlier study found a distance from the tumor to pleura of 14 mm as determined by pre-operative CT, to be the maximum tumor depth that can be imaged with an 800-nm fluorophore [18]. For SGM-101 (700 nm), this might be slightly lower [5, 19]. In the current study, five lesions had a distance to the pleura of more than 14 mm on CT, and none of these were fluorescent in vivo. A second application of intraoperative NIR fluorescence imaging is margin assessment. When close or positive resection margins are expected during surgery (e.g., when the tumor infiltrates the chest wall or a bronchus), intraoperative fluorescence imaging with SGM-101 may also be beneficial. For margin assessment, tumor depth is not influential. This is due to the fact that margin assessment is performed by imaging of the resection margin on the specimen on the back table. When positive margins are suspected, the wound bed can also be imaged to assess for residual signal. Given that 94% of the tumor bread loaves showed a positive fluorescence signal, it is expected that when tumor positive margins occur, they can be detected with this technique. Thus, patients with superficial nodules which are expected to be challenging to identify, or patients with tumors with potential tumor positive margins are most likely to benefit from the use of SGM-101.

A secondary objective of this study was to find the optimal dose of SGM-101 for the identification of CLM. For primary colorectal cancer, a dose of 10 mg was found to be the optimal dose [10]. Our study assessed three doses. In all dose groups, sufficient SBRs were found. SBRs appeared to decrease with increasing doses, but these differences were not significant. Therefore, a dose of 7.5 mg may be sufficient for pulmonary CLM imaging. The lowest dose is also preferable with regard to costs.

Recently, the first results were published on the use of SGM-101 for CLM and primary lung tumors by Azari et al. [20]. In this study, ten patients were included, of which four had CLM. A dose of 10 mg of SGM-101 was administered according to the standard dose for primary or recurrent colorectal cancer. In the paper, only SBRs from the closed-field imaging were reported. When comparing SBRs from this trial to our results we find similar results, with mean SBRs of 3–4. Findings of the present study build upon the work of Azari et al. by studying a larger amount of colorectal lung metastases. Moreover, we report the TBRs of the in vivo undissected lesions, presenting a realistic perspective on the current in-vivo imaging of CLM with SGM-101. Additionally, based on our results, a dose of 7.5 mg appears to be sufficient, as opposed to the previously suggested 10 mg, which could reduce costs and potential adverse events. For primary lung cancer surgery, several trials have been performed with other fluorescent tracers [4]. OTL-38 is a folate-α targeted fluorescent tracer for pulmonary adenocarcinoma that has been used in several studies for intraoperative imaging of primary lung adenocarcinoma. However, OTL-38 is not a good candidate for imaging of most other adenocarcinomas, including colorectal cancers. Less than 30% of the colorectal cancers express folate-α, while CEA is expressed on 95% of tumors [7, 21, 22]. In line with findings in primary colorectal cancer, our study demonstrated that CEA expression in colorectal lung metastases is high and independent of the serum CEA level. This confirms that serum CEA levels do not have to considered to select the optimal patients for molecular imaging with SGM-101 [23].

Several limitations of this study can be mentioned. The low number of patients might have affected dose finding. In addition, patients were not selected based on tumor location and distance to the pleura. This may explain why several nodules were not fluorescent when imaged intraoperatively. However, as we asked all eligible patients for participation, we most likely included a clinically representative cohort of patients.

In conclusion, the present study demonstrates the potential of fluorescence imaging of CLM with SGM-101. Closed-field imaging of bread loaves showed excellent targeting of the tracer to the tumor nodules, with maximum target expression on all tumor nodules. Challenges remain with in vivo detection of this tracer. Improving minimally invasive fluorescence imaging systems and optimal patient selection most likely enables the optimal efficacy of SGM-101 for CLM surgery.

### Supplementary information

Below is the link to the electronic supplementary material.

Figure S1 (PNG 7210 kb)

High Resolution (EPS 11266 kb)

## Data Availability

Upon reasonable request and in consultation with the sponsor.

## References

[1] Meyer Y, Olthof PB, Grünhagen DJ, de Hingh I, de Wilt JHW, Verhoef C, et al. Treatment of metachronous colorectal cancer metastases in the Netherlands: a population-based study. Eur J Surg Oncol. 2021;2:S742. 10.1016/j.ejso.2021.12.004 34895970

[2] Riihimäki M, Hemminki A, Sundquist J, Hemminki K. Patterns of metastasis in colon and rectal cancer. Sci Rep. 2016;6:29765. 27416752 10.1038/srep29765 PMC4945942

[3] Hao Z, Parasramka S, Chen Q, Jacob A, Huang B, Mullett T, et al. Neoadjuvant versus adjuvant chemotherapy for resectable metastatic colon cancer in non-academic and academic programs. Oncologist. 2022;28:48–58. 10.1093/oncolo/oyac209 PMC9847538 36200844

[4] Neijenhuis LKA, de Myunck L, Bijlstra OD, Kuppen PJK, Hilling DE, Borm FJ, et al. Near-infrared fluorescence tumor-targeted imaging in lung cancer: a systematic review. Life (Basel). 2022;12:446. 35330197 10.3390/life12030446 PMC8950608

[5] Keereweer S, Van Driel PB, Snoeks TJ, Kerrebijn JD, Baatenburg de Jong RJ, Vahrmeijer AL, et al. Optical image-guided cancer surgery: challenges and limitations. Clin Cancer Res. 2013;19:3745–54. 23674494 10.1158/1078-0432.CCR-12-3598

[6] Gutowski M, Framery B, Boonstra MC, Garambois V, Quenet F, Dumas K, et al. SGM-101: An innovative near-infrared dye-antibody conjugate that targets CEA for fluorescence-guided surgery. Surg Oncol. 2017;26:153–62. 28577721 10.1016/j.suronc.2017.03.002

[7] Tiernan JP, Perry SL, Verghese ET, West NP, Yeluri S, Jayne DG, et al. Carcinoembryonic antigen is the preferred biomarker for in vivo colorectal cancer targeting. Br J Cancer. 2013;108:662–7. 23322207 10.1038/bjc.2012.605 PMC3593555

[8] Meijer RPJ, de Valk KS, Deken MM, Boogerd LSF, Hoogstins CES, Bhairosingh SS, et al. Intraoperative detection of colorectal and pancreatic liver metastases using SGM-101, a fluorescent antibody targeting CEA. Eur J Surg Oncol. 2021;47:667–73. 33158638 10.1016/j.ejso.2020.10.034

[9] Schaap DP, de Valk KS, Deken MM, Meijer RPJ, Burggraaf J, Vahrmeijer AL, et al. Carcinoembryonic antigen-specific, fluorescent image-guided cytoreductive surgery with hyperthermic intraperitoneal chemotherapy for metastatic colorectal cancer. Br J Surg. 2020;107:334–7. 31960953 10.1002/bjs.11523 PMC7079046

[10] de Valk KS, Deken MM, Schaap DP, Meijer RP, Boogerd LS, Hoogstins CE, et al. Dose-finding study of a CEA-targeting agent, SGM-101, for intraoperative fluorescence imaging of colorectal cancer. Ann Surg Oncol. 2021;28:1832–44. 33034788 10.1245/s10434-020-09069-2 PMC7892528

[11] Hoogstins CES, Boogerd LSF, Sibinga Mulder BG, Mieog JSD, Swijnenburg RJ, van de Velde CJH, et al. Image-guided surgery in patients with pancreatic cancer: first results of a clinical trial using SGM-101, a novel carcinoembryonic antigen-targeting, near-infrared fluorescent agent. Ann Surg Oncol. 2018;25:3350–7. 30051369 10.1245/s10434-018-6655-7 PMC6132431

[12] Boogerd LSF, Hoogstins CES, Schaap DP, Kusters M, Handgraaf HJM, van der Valk MJM, et al. Safety and effectiveness of SGM-101, a fluorescent antibody targeting carcinoembryonic antigen, for intraoperative detection of colorectal cancer: a dose-escalation pilot study. Lancet Gastroenterol Hepatol. 2018;3:181–91. 29361435 10.1016/S2468-1253(17)30395-3

[13] Vahrmeijer AL. SGM-101 in locally advanced and recurrent rectal cancer (SGM-LARRC). ClinicalTrial.gov. Clinical Trials GOV ID: NCT04642924

[14] Vahrmeijer AL. Performance of SGM-101 for the delineation of primary and recurrent tumor and metastases in patients undergoing surgery for colorectal cancer. ClinicalTrials.gov. Clinical Trials GOV ID: NCT03659448

[15] Azargoshasb S, Boekestijn I, Roestenberg M, KleinJan GH, van der Hage JA, van der Poel HG, et al. Quantifying the impact of signal-to-background ratios on surgical discrimination of fluorescent lesions. Mol Imaging Biol. 2022;22:e186-95. 10.1007/s11307-022-01736-y PMC9971139 35711014

[16] Lauwerends LJ, van Driel P, Baatenburg de Jong RJ, Hardillo JAU, Koljenovic S, Puppels G, et al. Real-time fluorescence imaging in intraoperative decision making for cancer surgery. Lancet Oncol. 2021;22:e186–95. 33765422 10.1016/S1470-2045(20)30600-8

[17] Linders D, Deken M, van der Valk M, Tummers W, Bhairosingh S, Schaap D, et al. CEA, EpCAM, αvβ6 and uPAR expression in rectal cancer patients with a pathological complete response after neoadjuvant therapy. Diagnostics (Basel). 2021;11:516. 33799475 10.3390/diagnostics11030516 PMC8002064

[18] Okusanya OT, Holt D, Heitjan D, Deshpande C, Venegas O, Jiang J, et al. Intraoperative near-infrared imaging can identify pulmonary nodules. Ann Thorac Surg. 2014;98:1223–30. 25106680 10.1016/j.athoracsur.2014.05.026 PMC4185369

[19] Kennedy GT, Azari FS, Chang A, Nadeem B, Bernstein E, Segil A, et al. Comparative experience of short versus long wavelength fluorophores for intraoperative molecular imaging of lung cancer. Ann Surg. 2022;276:711–9. 35837887 10.1097/SLA.0000000000005596 PMC9463092

[20] Azari F, Meijer RPJ, Kennedy GT, Hanna A, Chang A, Nadeem B, et al. Carcinoembryonic antigen-related cell adhesion molecule type 5 receptor-targeted fluorescent intraoperative molecular imaging tracer for lung cancer: a nonrandomized controlled trial. JAMA Netw Open. 2023;6: e2252885. 36705924 10.1001/jamanetworkopen.2022.52885 PMC10292762

[21] D’Angelica M, Ammori J, Gonen M, Klimstra DS, Low PS, Murphy L, et al. Folate receptor-α expression in resectable hepatic colorectal cancer metastases: patterns and significance. Mod Pathol. 2011;24:1221–8. 21572402 10.1038/modpathol.2011.82

[22] Chen CI, Li WS, Chen HP, Liu KW, Tsai CJ, Hung WJ, et al. High expression of folate receptor alpha (FOLR1) is associated with aggressive tumor behavior, poor response to chemoradiotherapy, and worse survival in rectal cancer. Technol Cancer Res Treat. 2022;21:15330338221141796. 36426547 10.1177/15330338221141795 PMC9703519

[23] Boogerd LSF, Vuijk FA, Hoogstins CES, Handgraaf HJM, van der Valk MJM, Kuppen PJK, et al. Correlation between preoperative serum carcinoembryonic antigen levels and expression on pancreatic and rectal cancer tissue. Biomark Cancer. 2017;9:1179299X1771001. 10.1177/1179299X17710016 PMC5437985 28579847

