# Supplementary figures and images for: Intraoperative molecular imaging of colorectal lung metastases with SGM-101: a feasibility study
Source: Eur J Nucl Med Mol Imaging. 2023 Aug 8;51(10):2970–9. doi: 10.1007/s00259-023-06365-3 (PMC11300526; doi:10.1007/s00259-023-06365-3)

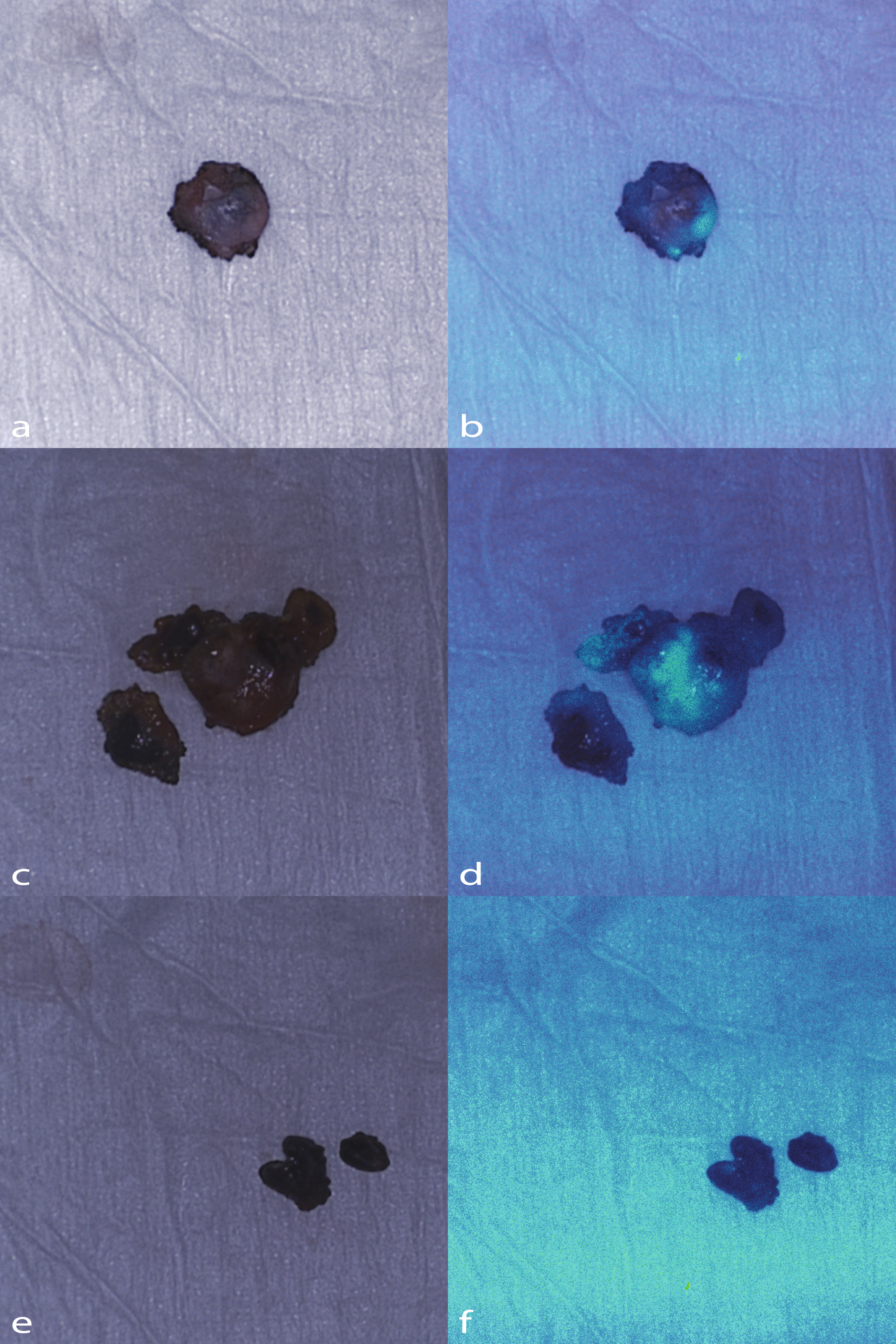

Supplement: Supplementary file 1 — (PNG 7210 kb) [file 259_2023_6365_Fig6_ESM.png]
